# Role of Extracellular Matrix-Mediated Interactions in Thymocyte Migration

**DOI:** 10.1155/2000/60247

**Published:** 2000

**Authors:** Wilson Savino, Sérgio Ranto Dalmau, Vinícius Cotta Dealmeida

**Affiliations:** 1 Laboratory on Thymus Research Department of Immunology Oswaldo Cruz Institute – Oswaldo Cruz Foundation Rio de Janeiro Brazil; 2 Department of Biochemistry Biology Institute University of the State of Rio de Janeiro and Program of Experimental Medicine Basic Research Center, National Cancer Institute Rio de Janeiro Brazil; 3 Laboratory of Cell Biology Department of Ultrastructure and Cell Biology Oswaldo Cruz Institute Oswaldo Cruz Foundation Rio de Janeiro Brazil; 4 Laboratory on Thymus Research Department of Immunology Oswaldo Cruz Institute Oswaldo Cruz Foundation Ave. Brasil 4365-Manguinhos - Rio de Janeiro 21045-900 Brazil

**Keywords:** thymus, fibronectin, laminin, integrins, lymphocytes, thymic epithelial cells

## Abstract

Cell adhesion, migration, differentiation and survival or death is amongst a large spectrum of biological responses that can be elicited by ligation of extracellular matrix components to their corresponding receptors. As regards the physiology of the thymus, cell migration is a crucial event in the general process of T cell differentiation. Studies on the intrathymic distribution of ECM components revealed that fibronectin, laminin and type IV collagen, are not restrictedly located at typical basement membrane sites, also forming a thick network in the medullary region of the thymic lobules, whereas very thin ECM fibers are found within the cortex. These ECM components are essentially produced by thymic microenvironmental cells, which also drive thymocyte differentiation. Signals triggered by ECM are conveyed into thymocytes or microenvironmental cells through specific membrane receptors, and most of them belong to the integrin type, such as the VLA-3, VLA-4, VLA-5 and VLA-6. *In vitro* studies revealed that adhesion of thymocytes to thymic microenvironmental cells is mediated by extracellular matrix. Such an adhesion is preferentially done by immature thymocytes. Importantly, ECM-mediated interactions also govern the entrance and exit of thymocytes in the lymphoepithelial complexes named thymic nurse cells. Lastly, pathological conditions, including infectious and autoimmune diseases, in which changes of ECM ligands and receptors are observed, course with alterations in thymocyte migration and death. In conclusion, the fact that ECM can modulate traffic, differentiation, death and survival of normal thymocytes adds clues for understanding how ECM-mediated interactions behave in the thymus, not only in normal, but also in pathological conditions.


*Developmental Immunology*, 2000, Vol. 7(2-4), pp. 279-291
Reprints available directly from the publisher
Photocopying permitted by license only

© 2000 OPA (Overseas Publishers Association)
N.V. Published by license under the
Harwood Academic Publishers imprint,
part of the Gordon and Breach Publishing Group.
Printed in Malaysia

### Role of Extracellular Matrix-Mediated Interactions in Thymocyte Migration

WILSON SAVINO[a*], SÉRGIO RANTO DALMAU[b] and VINÍCIUS COTTA DEALMEIDA[c]

[a]*Laboratory on Thymus Research, Department of Immunology, Oswaldo Cruz Institute – Oswaldo Cruz Foundation, Rio de Janeiro, Brazil,* [b]*Department of Biochemistry, Biology Institute, University of the State of Rio de Janeiro and Program of Experimental Medicine, Basic Research Center, National Cancer Institute, Rio de Janeiro, Brazil; and* [c]*Laboratory of Cell Biology, Department of Ultrastructure and Cell Biology, Oswaldo Cruz Institute, Oswaldo Cruz Foundation, Rio de Janeiro – Brazil*

Cell adhesion, migration, differentiation and survival or death is amongst a large spectrum of biological responses that can be elicited by ligation of extracellular matrix components to their corresponding receptors. As regards the physiology of the thymus, cell migration is a crucial event in the general process of T cell differentiation. Studies on the intrathymic distribution of ECM components revealed that fibronectin, laminin and type IV collagen, are not restrictedly located at typical basement membrane sites, also forming a thick network in the medullary region of the thymic lobules, whereas very thin ECM fibers are found within the cortex. These ECM components are essentially produced by thymic microenvironmental cells, which also drive thymocyte differentiation. Signals triggered by ECM are conveyed into thymocytes or microenvironmental cells through specific membrane receptors, and most of them belong to the integrin type, such as the VLA-3, VLA-4, VLA-5 and VLA-6. *In vitro* studies revealed that adhesion of thymocytes to thymic microenvironmental cells is mediated by extracellular matrix. Such an adhesion is preferentially done by immature thymocytes. Importantly, ECM-mediated interactions also govern the entrance and exit of thymocytes in the lymphoepithelial complexes named thymic nurse cells. Lastly, pathological conditions, including infectious and autoimmune diseases, in which changes of ECM ligands and receptors are observed, course with alterations in thymocyte migration and death. In conclusion, the fact that ECM can modulate traffic, differentiation, death and survival of normal thymocytes adds clues for understanding how ECM-mediated interactions behave in the thymus. not only in normal, but also in pathological conditions.

*Keywords:* thymus, fibronectin, laminin, integrins, lymphocytes, thymic epithelial cells

Extracellular matrix (ECM) represents a functional component, in continuous interactions with cells of all metazoa. Accordingly, various ECM receptors in a large variety of cell types have been cloned, and signal transduction pathways that are triggered when such receptors are activated by their ligands, have been described. Cell adhesion, migration, differentiation and survival or death are amongst a large spectrum of biological responses that can be elicited by ligation of a given ECM component to its corresponding receptor. As regards the physiology of one particular organ of the immune system, the thymus gland, a complete understanding of the role of ECM is far from being achieved. Nonetheless, a series of evi-

* Correspondence address: Wilson Savino, Laboratory on Thymus Research, Department of Immunology, Oswaldo Cruz Institute – Oswaldo Cruz Foundation, Ave. Brasil 4365 – Manguinhos, 21045–900 – Rio de Janeiro – BRAZIL. Phone: (55) (21) 280.1486 -- (55) (21) 598.4327, Fax: (55) (21) 280.1589 -- (55) (21) 590.3545, e-mail: savino@gene.dbbm.fiocruz.br -- savino@ioc.fiocruz.br dence further discussed below, does point to a functional relevancy of ECM glycoproteins in thymocyte migration. Additionally, we will discuss herein data suggesting that pathological changes of thymic ECM, as for example those induced by radiation or acute infections, might cause defects of intrathymic T cell migration, including their exit from the organ.

### INTRATHYMIC DISTRIBUTION OF EXTRACELLULAR MATRIX LIGANDS

The thymus is a primary lymphoid organ, in which bone marrow-derived T cell precursors undergo a complex process of maturation, eventually leading to the exit of positively selected thymocytes. Most immature thymocytes (bearing the phenotypes $CD3^-CD4^-CD8^-$ and $CD3^{low}CD4^+CD8^+$) are cortically located, whereas $CD3^{high}CD4^+CD8^-$ and $CD3^{high}CD4^-CD8^+$ mature cells are found in the medulla. These medullary single positive cells will further cross the blood vessel walls and leave the organ to colonize the periphery of the immune system (Anderson et al, 1996). Cell migration is thus a key event in the general process of intrathymic T cell differentiation. Importantly, thymocyte maturation is essentially driven by the thymic microenvironment; a tridimensional network comprised by epithelial cells and to a much lesser extent, dendritic cells, phagocytes, fibroblasts and ECM moieties. The thymic epithelial cell (TEC) network, is a heterogeneous tissue in terms of both morphology and phenotype. One lymphoepithelial complex, the thymic nurse cell complex (TNC) has been isolated *ex vivo*, and corresponds to a multicellular structure formed by one TEC, that in mice can harbor 20–200 thymocytes (Wekerle and Ernst, 1980). These TNC complexes are located in the cortex of the thymic lobules (van Ewijk, 1988; Li et al, 1998). Accordingly, most intra-TNC thymocytes bear the $CD4^+CD8^+$ double positive phenotype (Li et al, 1992; Lahoud et al, 1992), although immature double negative as well as mature single positive cells can be found. Actually, TNCs may represent a special microenvironment for thymocyte differentiation. In this complex distinct interactions have been evidenced, including those mediated by major histocompatibility complex products/T cell receptor, soluble products such as cytokines and thymic hormones), as well as extracelllular matrix (see review Villa-Verde et al, 1995).

The distribution of ECM in the normal thymus does not follow a homogenous pattern, and glycoproteins such as fibronectin, laminin and type IV collagen are not restrictedly located at typical basement membrane sites. In the medullary region of the thymic lobules, they form a rather thick medullary network, whereas very thin ECM fibres are found within the cortex (Fig. 1). This is in contrast with the tenascin distribution profile, virtually concentrated in the medulla and cortico-medullary junction (Oklind et al, 1993; Freitas et al, 1995). The distribution of type I collagen is also distinct, being limited to the capsule, intraseptal and perivascular spaces (Berrih et al, 1985). It is noteworthy that the intrathymic pattern of various ECM glycoproteins is largely conserved in mammals (Meirelles de Souza et al, 1993), suggesting a relevant role of these molecules in thymus physiology.

Distinct cell types are able to produce ECM components. We showed that cultured TEC can produce laminin, fibronectin and type IV collagen, as revealed in various human and murine TEC preparations, including TNC (Berrih et al, 1985; Lannes-Vieira et al, 1991; Meirelles de Souza and Savino, 1993; Villa-Verde et al, 1994). Further recent results suggest that these ECM components can also be produced by fibroblasts as well as phagocytic cells of the thymic reticulum (Anderson et al, 1997; Ayres Martins et al, submitted). Production of type I collagen is likely to be restrictedly secreted by typical fibroblasts (Berrih et al, 1985; Lannes-Vieira et al, 1991), whereas tenascin seems to be produced by yet unidentified nonepithelial microenvironmental cells, possibly pericytes (Freitas et al, 1995).

In the last few years, isoforms of ECM glycoproteins have been reported in the thymus. It has been shown that classical fibronectin, which is recognized by VLA-5 through the RGD motif, is located throughout the thymic parenchyma, whereas the isoform derived from alternative splicing of the fibronectin mRNA, and that is recognized by VLA-4 is restricted

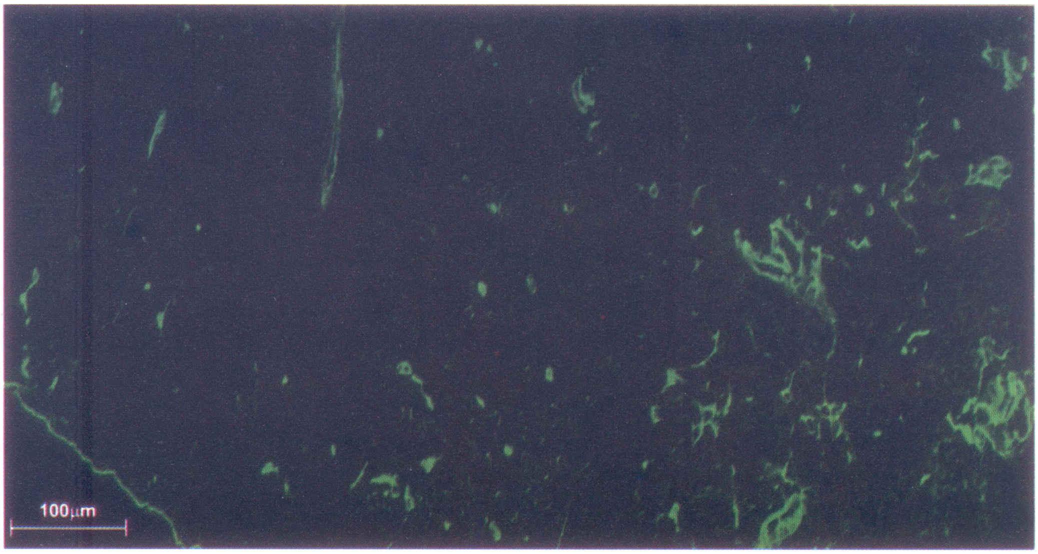

FIGURE 1 Intrathymic distribution of fibronectin, as revealed by confocal microscopy. Note that the fibronectin-containing network is denser in the medulla (right part on the panel) of the thymic lobule, as compared to the cortex (left part on the panel). Additionally, fibronectin is seen around blood vessels. Bar = 100 µm (see Color Plate XVIII at the back of this issue)

to the medulla, clearly defining the cortico-medullary junction of the thymic lobules (Crisa et al, 1996).

Additionally, laminin isoforms, generated by transcription of distinct gene's, and not by mRNA alternative splicings, have been reported. The first isoform characterized in the mouse thymus was laminin-2 (also named merosin), formed by the heterotrimer $\alpha2\beta1\gamma1$ (Chang et al, 1993, 1995). These authors showed that thymocytes bind this isoform. Interestingly, an aberrant thymocyte development was recently detected in the dy/dy mutant mouse, which lacks laminin-2 (Magner et al, 2000). Such alterations included thymic atrophy with decreased relative numbers of $CD4^+CD8^+$ thymocytes, and an increase of apoptosis in the $CD4^-CD8^-$ cells.

In addition, laminin 5 (the heterotrimer $\alpha3\beta3\gamma2$) was evidenced in the human thymus, being able to trigger outside-in signals to thymocytes (Vivinus-Nebot et al, 1999). In fact, by RT-PCR analysis we recently found several laminin isoforms in human thymic epithelial cell preparations (Ocampo et al, submitted). Although it remains to be determined whether all isoforms are functional in the physiology of the thymus, we defined that laminin-1 and laminin-2 can modulate thymocyte migration within TNC complexes. It is noteworthy that in the human thymus, laminin-2 is rather restricted to cortical epithelial cells (Ocampo et al, submitted), whereas laminin-5 is located surrounding small blood vessels (Mizushima et al, 1998).

Expression of proteoglycans and glycosaminoglycans by thymic cells is less studied. Pioneer *in vitro* work (Britz et al, 1983) revealed the production of sulfated glycosaminoglycans by the thymic microenvironment and thymocytes. Much more recently, we showed that TEC could secrete hyaluronic acid, as well as a heparan sulfate bearing short but highly sulfated regions (Werneck et al, 1999). This is in keeping with previous data showing hyaluronic acid in human thymus frozen sections (Patel et al, 1995), and the fact that hyaluronic acid plays a role in thymocyte maturation. Such an effect was actually seen using thymus fetal organ culture systems: treatment of fibroblast + epithelial cell reagreggates impaired the *in vitro* differentiation of immature $CD25^+CC44^+$ thymocytes (Anderson et al, 1997).

*In vitro* production of ECM glycoproteins and glycosaminoglycans by distinct thymic microenvironmental cells is summarized in Table I.

TABLE I *In vitro* Expression of Extracellular Matrix Glycoproteins and Glycosaminoglycans by Thymic Microenvironmental Cells[a]

| *Extracellular matrix molecules* | *Epithelial cells* | *Phagocytic cells* | *Fibroblasts* |
|---|---|---|---|
| Fibronectin | • | • | • |
| Laminin | • | • | • |
| Type IV collagen | • | • | • |
| Type I collagen | o | o | • |
| Tenascin | o | o | o |
| Hyaluronic acid | • | nd | nd |
| Heparan sulfate | • | nd | nd |

a. presence (•); absence (o); not determined (nd).

### EXTRACELLULAR MATRIX RECEPTORS ARE EXPRESSED BY BOTH THYMOCYTES AND MICROENVIRONMENTAL CELLS

The biological effects of ECM components upon the various thymic cell types occur via interaction with specific membrane receptors expressed by these cells. Many of them belong to the integrin family, corresponding to transmembrane αβ heterodimers able to provide cell adhesion and intracellular signalling. Several groups including ours showed that TEC express fibronectin and laminin receptors, respectively VLA-5 (α5β1) and VLA-6 (α6β1) integrins (Giunta et al, 1991; Lannes-Vieira et al, 1993; Villa-Verde et al, 1994). Additionally, the VLA-4 (α4β1) fibronectin receptor has been reported in human thymic epithelial cells (Nieto et al, 1996). Interestingly, confocal microscopy analyses conducted in the human thymus showed that integrins were polarized on TEC at discrete locations: α6β4 lined the basal surface of TEC monolayers, whereas α3β1 was mostly found at TEC-TEC contacts. Moreover, it was noteworthy that the density of both receptors was highly enhanced at the boundaries with adherent thymocytes (Ramarli et al, 1998).

Thymocytes also express VLA-4 and VLA-5 fibronectin receptors, with the former being highly expressed in CD4/CD8 double-negative cells (Sawada et al, 1992; Salomon et al, 1997; Dalmau et al, 1999). As regards laminin receptors, it was showed that the majority of mouse fetal thymocytes expresses the integrin α6β4. Nonetheless, this receptor is developmentally down regulated, so that most adult thymocytes express the classical VLA-6 laminin receptor (Wadsworth et al, 1992), as well as the VLA-3 (α 3β1) integrin (Chang et al, 1995). Yet, the α6β4 expression restricted to $CD4^{-}CD8^{-}$ cells was recently argued by the data showing this receptor in mature medullary located human thymocytes (Vivinus-Nebot et al, 1999).

Typical cytofluorometric profiles depicting the expression of VLA-4, VLA-5 and VLA-6 by murine thymocytes are illustrated in figures 2 and 3.

One of the first well-characterized ECM receptor in the thymus is the proteoglycan CD44, and its expression on thymocytes is particularly higher in very immature cells, thus serving as a marker for early differentiating thymocytes (Lynch and Ceredig, 1988). CD44 is a hyaluronic acid receptor, but it can also bind to fibronectin and collagen. This receptor has been determined in the human thymus, bearing a distribution similar to that of fibronectin (Patel et al, 1995). Interestingly, CD44 isoforms have been identified in the human thymus, and may reflect specific functions in thymocyte differentiation (Haynes et al, 1989; Patel et al, 1995). In terms of microenvironmental cells, we and others demonstrated that CD44 is also expressed in cultured TEC and more recently in phagocytic cells (Villa-Verde et al, 1994; Patel et al, 1995; Oliveira-dos-Santos et al, 1997). Interestingly, a distinct hyaluronic acid receptor, exclusively present on thymocytes, has been described in the human thymocytes, and its expression is regulated by TEC (Pilarski et al, 1993).

Conjointly, the expression of ECM receptors on thymocytes and microenvironmental cells, together with the intrathymic production of distinct ECM components, provide a molecular basis for the existence of heterocellular ECM-mediated interactions in the organ.

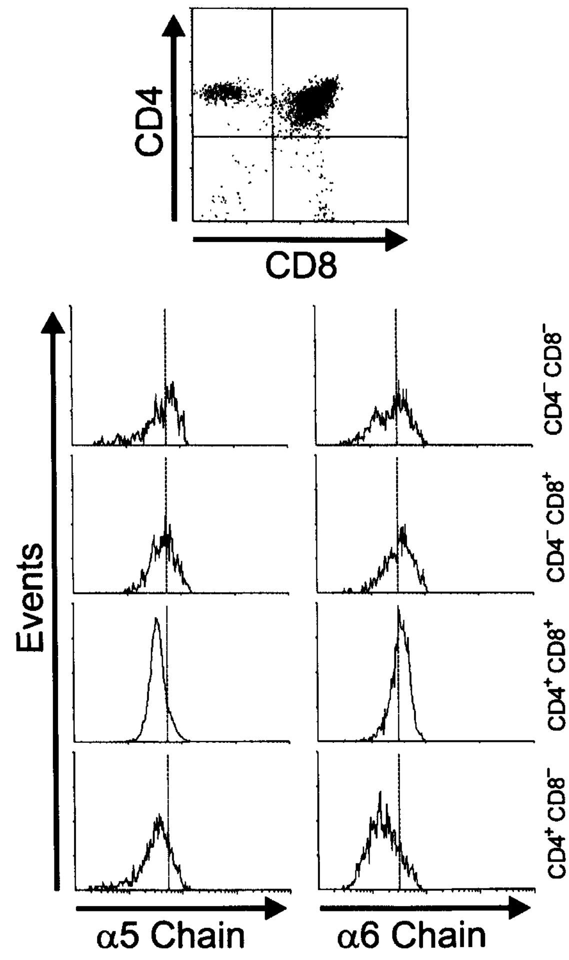

FIGURE 2 Expression of α5 and α6 integrin chains in thymocyte subsets of C57B1/6 young mice. The quadrants in the dot plot define the $CD4^+CD8^-$ (upper left), $CD4^+CD8^+$ (upper right), $CD4^-CD8^-$ (lower left), and $CD4^-CD8^+$ (lower right) thymocytes shown in histograms. Vertical bars evidence cells with high integrin expression

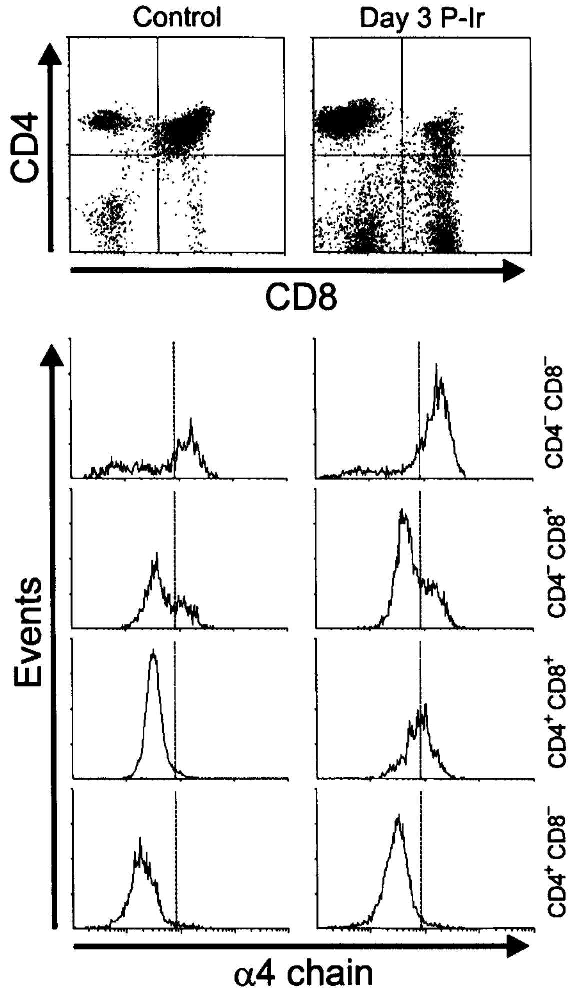

FIGURE 3 Expression of α4 integrin chain in CD4/CD8-defined thymocyte subsets, obtained from control (non-irradiated) or day 3 following 250 cGy whole body γ-irradiation. Vertical bars evidence cells with high integrin expression. Note the high density of VLA-4 in $CD4^-CD8^-$ immature cells

### EXTRACELLULAR MATRIX DEPENDENT THYMOCYTE MIGRATION THROUGH THYMIC NURSE CELLS

*In vitro*, TNCs release thymocytes through an active process, depending on metabolism and cytoskeleton integrity (Andrew and Boyd, 1985). Additionally, TNC-derived epithelial cells can reconstitute lymphoepithelial complexes after being co-cultured with fetal thymocytes, thus placing TNC as an *in vitro* model of thymocyte migration (reviewed by Villa-Verde et al, 1995).

We showed that thymocyte release from TNC complexes is enhanced by fibronectin and laminin and diminished by the corresponding antibodies (mAb) or, in the case of fibronectin, antagonist peptides. In addition, anti-fibronectin, anti-VLA-5, anti-laminin or anti-VLA-6 antibodies significantly abrogate the exit of thymocytes from TNCs. Similar blocking effects of anti-ECM or anti-ECM receptors reagents can be seen in the *de novo* formation of TNC complexes by mixing TNC-derived epithelial cells plus fetal thymocytes (Villa-Verde et al, 1994). These data indicate that ECM-mediated TEC/thymocyte interactions play a role in the traffic of thymocytes within TNCs, affecting both the entrance and exit of the lymphocytes in this particular microenvironmental niche.

Yet, the above results raise a paradox that remains to be fully elucidated: when blocking interactions mediated by adhesive proteins, such as laminin or fibronectin, migration is stopped. This may be explained by the fact the migration needs adhesion → de-adhesion repeatedly. Then if we abrogate any of these "*walking steps*", migration as a whole is blocked. Perhaps this accounts to explain why focal adhesion kinase in thymocytes is highly and constitutively expressed in its activated (thyrosine phosphorilated) state in all thymocyte subsets; deactivation being induced when mAb with specificity for $\beta1$, $\alpha4$, LFA-1 or $\alpha_L$integrins bind to thymocytes (Kanazawa et al, 1995).

Another pathway for inducing de-adhesion, and which remains to be evaluated, refers to whether metalloproteinases are triggered in these conditions and if play a role thymocyte migration, inside and/or outside TNCs. Lastly, but not mutually exclusive, de-adhesion may be also influenced by de-adhesive ECM molecules, such as tenascin and galectin-3. In fact, we recently demonstrated the expression of galectin-3 by thymic microenvironmental cells, as well as its positive role on de-adhesion of thymocytes from TEC and thymic phagocytic cells (Villa-Verde et al, 1999).

### THYMOCYTE ADHESION TO THYMIC MICROENVIRONMENTAL CELLS IS AN ECM-MEDIATED EVENT

One of the essential events in cell migration is the tandem adhesion → de-adhesion events of the migrating cells onto the substrate. We demonstrated that adhesion of thymocytes to cultured TEC line is enhanced in the presence of ECM components, such as fibronectin and/or laminin, whereas antibodies against these adhesive molecules promote an opposite effect. Moreover, pre-treatment of TEC with anti-VLA-5 or anti-VLA-6 antibodies promoted the same effects (Lannes-Vieira et al, 1993; Lagrota-Cândido et al, 1996; Mello-Coelho et al, 1997). Similar findings were obtained when thymocytes were led to adhere onto primary cultures of TNC-derived thymic epithelial cells (Villa-Verde et al, 1994). Additionally, it has been shown that thymocytes can directly bind to fibronectin as well as laminin (Utsumi et al, 1991; Sawada et al, 1992; Chang et al, 1993, 1993; Vivinus-Nebot et al, 1999).

Concerning thymocytes, it has been shown that very immature cells use VLA-4 to adhere and migrate onto fibronectin, whereas mature cells apparently use both VLA-4 and VLA-5 to migrate (Salomon, et al, 1994; Crisa et al, 1996).

Functional studies performed with mAbs anti-$\beta1$ and -$\beta4$ integrin monoclonal antibodies (mAb) showed that $\beta1$, and, to a much lower extent, $\beta4$ heterodimers are involved in the TEC-thymocyte adhesion. Thymocyte contact or mAb-mediated ligation of $\alpha3$, $\alpha6$, $\beta1$, and $\beta4$ integrins was investigated as a potential inducer of intracellular signaling in TEC. Thymocyte adhesion or cross-linking of mAbs bound to integrins clustered at the TEC/thymocyte contact sites led to activation of interleukin-6 (IL-6) gene transcription factors, namely NF-IL6 serine phosphorylation and NF-κB nuclear targeting, as well as to increased IL-6 secretion (Ramarli et al, 1998).

In addition to TEC lines, it has been shown that immature thymocytes are able to adhere onto TNC-derived primary TEC cultures and that such an event is ECM-dependent (Villa-Verde et al, 1994).

Interestingly, thymocyte adhesion to cultured TEC seems to be down regulated by galectin-3 (Villa-Verde et al, 1999). Nonetheless, since galectin-3 binds adhesive ECM proteins, it remains to be defined whether the de-adhesive effect is due to direct binding on the corresponding receptor or secondary to spatial changes promoted in laminin and/or fibronectin, thus preventing adhesion.

Furthermore, it is conceivable that thymocyte/TEC de-adhesion also results from the production of metalloproteinases that would in turn breakdown ECM adhesive proteins, thus disrupting ECM/ ECM receptor ligation. This hypothesis has not been tested as well. Yet, at least metalloproteinase 9 (also named gelatinase B) has been detected in thymic microenvironmental cells (Aoudjit et al, 1997).

It should be noted that ECM-mediated thymocyte adhesion $\rightarrow$ de-adhesion is not probably restricted to epithelial cells. We evidenced that phagocytic cells of the thymic microenvironment also allow thymocyte adhesion through ECM ligands and receptors, such as fibronectin/VLA-4, fibronectin/VLA-5 and laminin/VLA-6 (Ayres-Martins et al, submitted).

### PREFERENTIAL BINDING OF IMMATURE THYMOCYTES TO THYMIC EPITHELIAL CELLS: LESSONS FROM SUBLETHALLY IRRADIATED MICE

In addition to the data discussed above, we used enriched populations of immature thymocytes to study their ability to adhere onto TEC monolayers, and to correlate with the expression of ECM receptors. Mice sublethally exposed to a single 250 cGy wholebody dose of $\gamma$-irradiation show a profound thymic collapse with thymocyte depletion as early as 24 following irradiation. The onset of regeneration occurs from day 2 to 3 post-irradiation, when we found a remarkable increase in the absolute numbers of $CD3^- CD25^{hi}CD44^+$ and $CD3^-CD25^{in/hi}CD44^-$ cells, together with higher expression of $\alpha$4 and $\alpha$5 integrin chains (Dalmau et al, 1999). This pattern is maintained until the $CD4^+CD8^+$ young stage is achieved. From this stage on, there is a downregulation of these receptors with the generation of $CD4^+$ or $CD8^+$ single positive thymocytes bearing intermediate levels $\alpha$4 and $\alpha$5 integrins, as illustrated in figure 3. Importantly, the increased expression of $\alpha$4 and $\alpha$5 chains in thymocytes was strongly correlated with their adhesiveness to thymic epithelial cells (TEC) *in vitro*.

To further address this issue, we used anti-$\alpha$4 or anti-$\alpha$5 chain mAb reported to block integrin binding to fibronectin, or peptides that mimic fibronectin moieties and interfere with their recognition by integrins. Adhesion was strongly blocked by anti-$\alpha$4 chain mAb and the fibronectin 1–25 IIICS peptide, which contains the critical LDV motif. By contrast, addition of the fibronectin 90–109 IIICS segment containing the REDV motif, also recognized by $\alpha$4 integrin, exhibited a poor inhibitory effect. Anti-$\alpha$5 chain mAb or fibronectin GRGDS peptide, which contains the RGD sequence recognized by $\alpha5\beta1$ integrin, exhibited no or very poor anti-adhesive action (Fig. 4). These findings are in keeping with those suggesting that in immature human thymocytes preferentially use VLA-4 to migrate, whereas mature thymocytes use both VLA-4 and VLA-5 fibronectin receptors (Crisa et al, 1996).

### ENTRANCE AND EXIT OF CELLS FROM THE THYMUS: ECM-MEDIATED EVENTS?

In addition to thymocyte migration within the thymic parenchyma, it is crucial to determine whether the entrance of bone marrow-derived precursors into the thymus, as well as the exit of mature thymocytes from the organ is governed by ECM-mediated interactions. Yet, little has been done in this respect. However, the few data available favor this hypothesis. In a series of blocking experiments *in vitro*, it has been shown that entrance of T cell precursors through thymic blood vessels can be abrogated with anti-CD44 or anti-VLA-6 antibodies (Ruiz et al, 1995). Additionally, the presence of anti-CD44 partially prevented the exit of *in vivo* fluorochrome-labeled thymocytes. It has been proposed that thymocyte exit from the organ occurs orderly, as if the cells were rolling on a conveyor belt (Scollay and Godffrey, 1995). We then postulated that such a conveyor belt is a supramolecular arrangement of ECM (synthesised by distinct microenvironmental cell types), that drives migration of differentiating thymocytes, ultimately allowing the exit of mature positively selected cells (Savino et al, 1996). This hypothetical molecular basis for thymocyte migration is illustrated in figure 5.

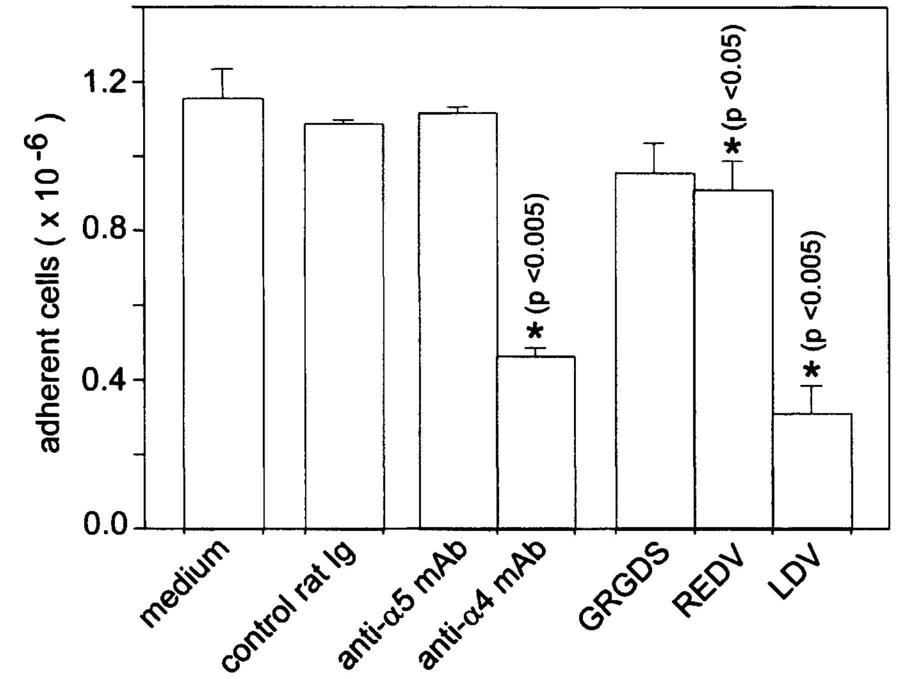

FIGURE 4 Adhesion of immature thymocytes from mice after day 3 post-irradiation to the thymic epithelial cell line 2BH4. Thymocytes ($1.5 \times 10^7$) were pre-incubated for 30 min. in medium alone, medium plus mAb directed to fibronectin receptors (or rat Ig as a negative control), or medium plus peptides mimicking fibronectin moieties, and then added to the 2BH4 TEC monolayers. After incubation for 1 h at 37 °C followed by washings with warm medium, adherent thymocytes were detached by beating the culture flasks and flushing the medium and counted. Antibodies were added at 20 μg/ml and peptides at 1 mg/ml

### DEFECTIVE THYMOCYTE MIGRATION IN MURINE CHAGAS DISEASE: POSSIBLE RELATIONSHIP WITH EXTRACELLULAR MATRIX

If ECM-mediated interactions are relevant to normal thymocyte migration, one can predict that abnormal expression of ECM ligands and/or receptors can lead to defects in thymocyte migration. One particular case in which an enhancement of these molecules occurs is the murine infection by the protozoan parasite *Trypanosoma cruzi*, the causative agent of Chagas disease. In the acute phase of this infection, we showed a severe thymic atrophy with depletion of $CD4^+CD8^+$ cortical thymocytes, which undergo massive apoptosis (Savino et al, 1989; Leite-de-Moraes et al, 1992). In parallel, there is a progressive enhancement of intralobular ECM ligands, including fibronectin, type IV collagen and laminin. These alterations are accompanied by changes in the expression of corresponding receptors VLA-4, VLA-5 and VLA-6 on the remaining thymocytes (Fig. 6).

In addition to these *in vivo* data, cultured TNC, infected either *in vitro* or derived from infected mice exhibited increased ECM contents. This correlated with an enhancement of thymocyte release from these lymphoepithelial complexes (Cotta-de-Almeida et al, 1997).

The abnormal traffic of intra-TNC thymocytes, together with the intrathymic enhancement of ECM and ECM receptors by thymocytes and TEC suggest that the exit of these cells may also be affected. In fact, we found a rise in the percentages of $CD4^+CD8^+$ cells in peripheral lymphoid organs in acutely infected animals (Cotta de Almeida et al, submitted to publication). As ascertained in studies using Balb/c

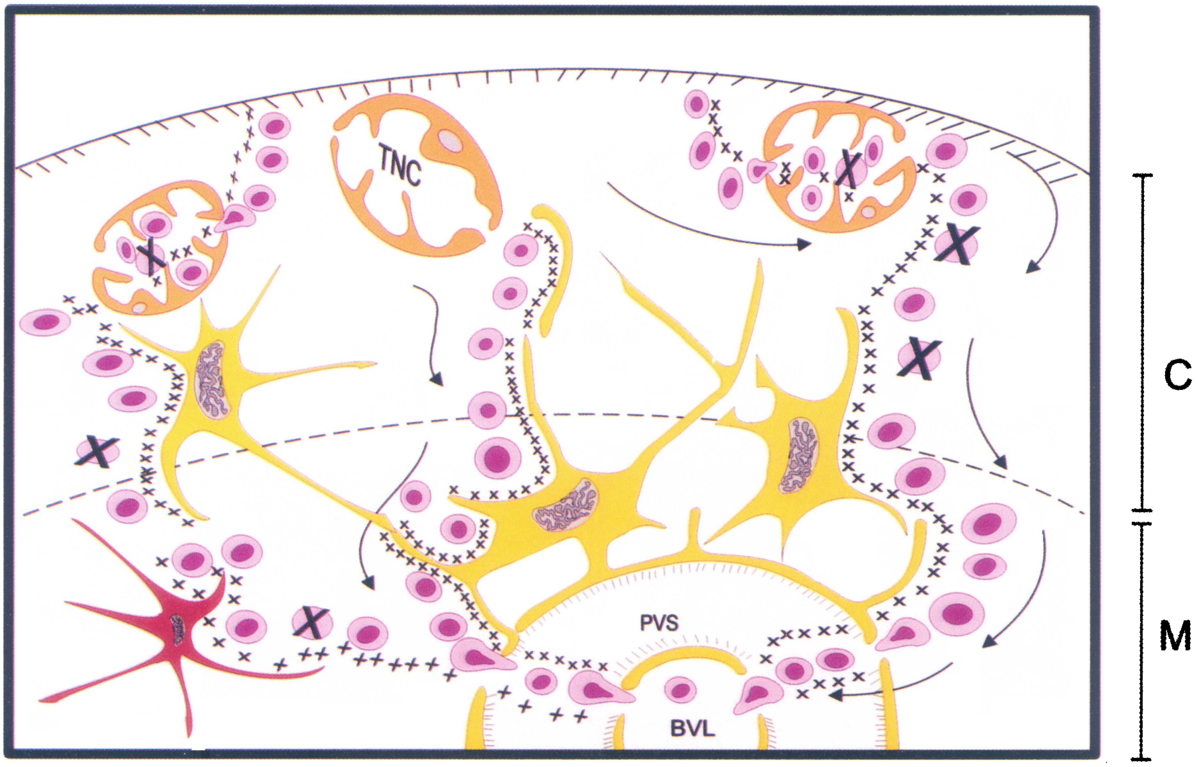

FIGURE 5 The conveyor belt hypothesis for intrathymic T cell migration: role of extracellular matrix. Accordingly, thymocyte migration follows an ordered pattern so that those thymocytes firstly generated and positively selected, are the first to leave the organ, as they were migrating on a conveyor belt. The molecular basis for such conveyor belt can be a macromolecular arrangement formed by a variety of ECM components, which are tridimensionally structured as a result of ligation with receptors expressed on microenvironmental cells (that also produce such ECM elements). Thymocytes then migrate onto such tridimensional structure, also using their ECM receptors. Along with such ECM-driven migration, thymocytes can encounter and interact with distinct microenvironmental cell types, including thymic nurse cells (TNC), other cortical and medullary epithelial cells (labeled in yellow), as well as dendritic cells (red). Positively selected thymocytes then cross the perivascular spaces (PVS) and reach the interior of the blood vessels (BV), thus leaving the thymus (see Color Plate XIX at the back of this issue)

mice, part of these T cells express TCR Vβ segments, such as Vβ5 and Vβ12, which that are normally present only in the thymus prior to negative selection. Theoretically, these lymphocytes may be at the origin of anti-myocardial cell autoreactivity mediated by $CD4^+$ T lymphocytes reported in the chronic phase of the disease (Ribeiro dos Santos et al, 1992), and that also use ECM-mediated interactions to migrate (Silva-Barbosa et al, 1997).

In any case, the escape of immature cells may be related to the disturbed intrathymic T cell migration in these animals. Supporting this hypothesis is the fact that, such $CD4^+CD8^+$ lymphocytes abnormally found in the periphery exhibited high densities of VLA-4, VLA-5 and VLA-6 (Cotta de Almeida et al, submitted).

### IS THERE AN EXTRACELLULAR MATRIX-RELATED DEFECT OF THYMOCYTE MIGRATION IN NONOBESE (NOD) MICE?

Abnormal thymocyte migration may also occur in autoimmune diseases. In the NOD mouse thymus we

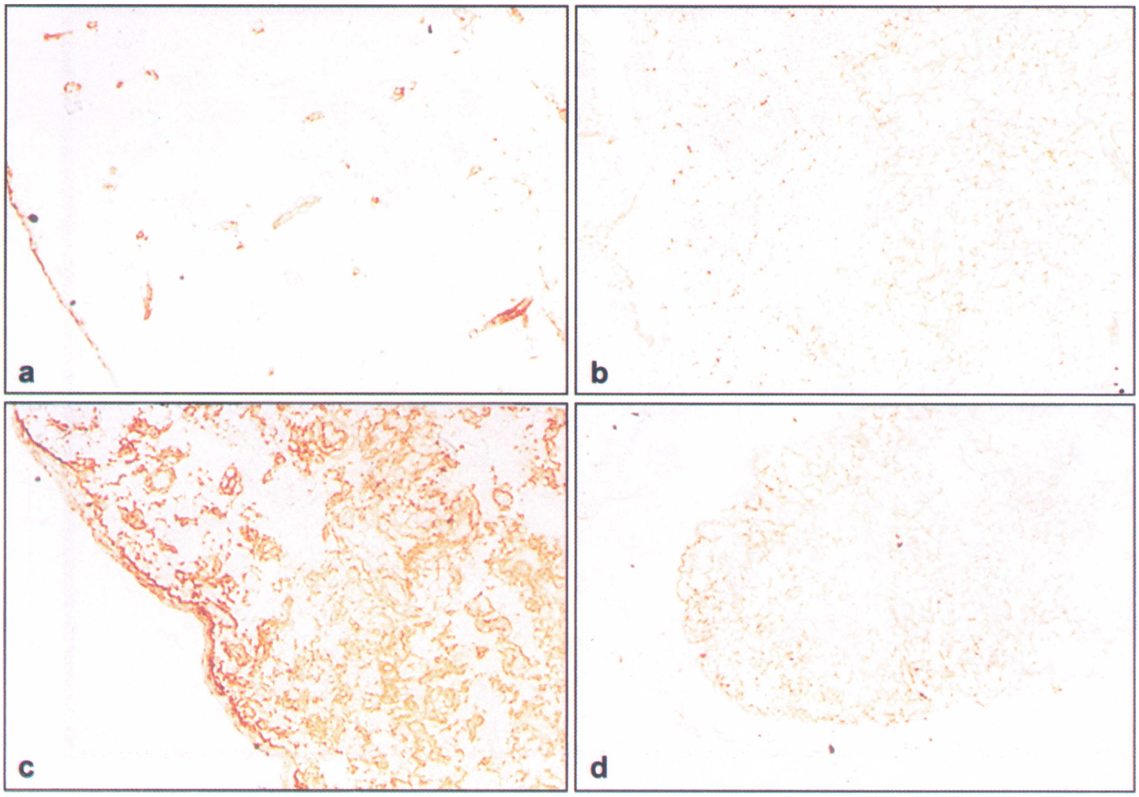

FIGURE 6 Enhancement of laminin and laminin receptor expression in the thymus of *Trypanosoma cruzi* acutely-infected mice. In these immunoperoxidase assays, panels **a** and **b** correspond to immunostainings for laminin and VLA-6 respectively, in the thymus from control animals. In thymuses from *T. cruzi* infected animals there is an increase in the amounts of both laminin and VLA-6 (respectively depicted in panels **c** and **d**). x450 (see Color Plate XX at the back of this issue)

previously demonstrated the formation of giant perivascular spaces (PVS), filled with mature thymocytes and an ECM-containing network (Savino et al, 1991; 1993). Such intrathymic alteration increases in size along with age, suggesting that thymocytes be gradually accumulated within the giant PVS. Actually, long term daily injection of bromodeoxyuridine revealed that thymocytes are somewhat arrested in these structures (our unpublished data). In the context of ECM-mediated thymocyte migration, we could predict a defect in the expression of ECM receptors by thymocytes and/or by thymic microenvironmental cells. In fact, it was reported VLA-5 decrease in mature thymocytes from the nonobese diabetic mouse thymus (Uniyal et al, 1998). Such a decrease is already seen in immature thymocytes, and is not restricted to the VLA-5 fibronectin receptor, but (to a lesser extent) it is also seen in as regards the VLA-4 receptor.

### CONCLUDING REMARKS AND OPEN QUESTIONS

The data reviewed above provide a strong evidence showing that the intrathymic expression of a variety of extracellular matrix components and corresponding receptors may be functionally linked to relevant events in thymus physiology, particularly thymocyte migration. Additionally, recent results although conflicting, suggest that thymocyte death and survival can be under the influence of extracellular matrix (Tchilian et al 1997). For the moment it seems that interaction with fibronectin via α4 integrins helps in TCR signaling for the positive selection (i.e. proliferation) of immature $CD4^-CD8^-$ cells (Halvorson et al 1998), whereas interaction with fibronectin via VLA-5 sensitizes for TCR signaling increasing negative selection (i.e. apoptosis) of $CD4^+CD8^+$ cells (Takayama et al, 1998).

A further putative function for thymic ECM is to concentrate some cytokines that might be relevant to intrathymic T cell differentiation. In this respect, is has been shown that IL-7, a key cytokine for early T cell differentiation in the thymic can bind to thymic ECM (Kitazawa et al, 1997). Furthermore, considering that interferon-γ can bind laminin (Hershkoviz et al, 1993; Li et al, 1998), it is easy to realize how important will be the knowledge of intrathymic cytokine-ECM interaction.

Lastly, since ECM can modulate traffic, death and survival of normal thymocytes, it is conceivable that the generation of the T cell repertoire can be influenced by ECM-mediated interactions, that may have a tuning function before, during and/or after the biological events of positive and negative selection. *In vivo* experiments as well as fetal thymus organ cultures may be helpful for testing this hypothesis.

In any case, the fact that ECM can modulate traffic, differentiation, death and survival of normal thymocytes adds clues for understanding how ECM-mediated interactions behave in the thymus, not only in normal, but also in pathological conditions.

### Acknowledgements

This work was partially supported with grants from Brazilan Minister of Health, CNPq, FAPERJ, FIOCRUZ, PADCT/CNPq and PRONEX/CNPq, and Ary Fauzino Foundation (Brazil).

### References

Anderson G., Moore N.C., Owen J.J.T., and Jenkinson E.J. (1996). Cellular interactions in thymocyte development. Ann. Rev. Immunol. 14:73–99.

Anderson G., Anderson K.L., Tchilian E.Z., Owen J.J., and Jenkinson E.J. (1997). Fibroblast dependency during early thymocyte development maps to the CD25+ CD44+ stages and involves interactions with fibroblast matrix molecules. Eur. J. Immunol. 27: 1197–1203.

Aoudjit F., Esteve P.O., Desrosiers M., Potworowski E.F., and St.-Pierre Y. (1997). Gelatinase B (MMP-9) production and expression by stromal cells in the normal and adult thymus and experimental thymic lymphoma. Int. J. Cancer 71:71–78.

Berrih S., Savino W., and Cohen S. (1985). Extracellular matrix of the human thymus. An immunofluorescence study on frozen sections and cultured thymic epithelial cells. J. Histochem. Cytochem. 33:655–664.

Britz J.S., and Hart G.H. (1983). Biosynthesis of glycosaminoglycans by epithelial and lymphocytic components of murine thymus. J. Immunol. 131:1420–1425.

Chang A.C., Wadsworth S., and Coligan J.L. (1993). Expression of merosin in the thymus and its interaction with thymocytes. J. Immunol. 151:1789–1794.

Chang A.C., Salomon D.R., Wadsworth S., Hong M.-J.P., Mojcik C.F., Otto S., Shevach E.M., and Coligan, J.E. (1995). α3β1 and α6α1 Integrins mediate Laminin/Merosin binding and function as costimulatory molecules for human thymocyte proliferation. J. Immunol. 154:500–510.

Cotta-de-Almeida V, Bertho AL, Villa-Verde DMS, and Savino W (1997). Phenotypic and functional analysis of thymic nurse cells following acute *Trypanosoma cruzi* infection. Clin. Immunol. Immunopathol. 82:125–132.

Crisa L., Cirulli V., Ellisman M.H., Ishii J.K., Elices M.J., amd Salomon D.R. (1996) cell adhesion and migration are regulated at distinct stages of thymic T cell development: the roles of fibronectin, VLA-4 and VLA-5. J. Exp. Med. 184:215–228.

Dalmau SR, Freitas CS & Savino W (1999). High expression of fibronectin receptors and L-selectin as a hallmark of early steps of thymocyte differentiation: lessons from sublethally irradiatad mice. Blood 93:974–990.

Freitas C.S., Lyra J.S.P.O., Dalmau S.R., and Savino W. (1995). *In vivo* and *in vitro* expression of tenascin by human thymic microenvironmental cells. Develop. Immunol. 4:139–147.

Giunta M., Fabre A., Ramarli D., Grossi C.E., and Corte G. (1991) A novel integrin involved in thymocyte-thymic epithelial cell interactions. J. Exp. Med., 173:1537–1544.

Halvorson M.J., Magner W., and Coligan J.E. (1998). α4 and α5 integrins costimulate the CD3-dependent proliferation of fetal thymocytes. Cellular. Immunol. 189:1–9.

Haynes B.F., Telen M.J., Hale L.P., and Denning S.M. (1989). CD44 – A molecule involved in leukocyte adherence and T cell activation. Immunol. Today, 10:423–436.

Hershkoviz R., Gilat D., Miron S., Mekori Y.A., Aderka D., Wallach D., Vlodavsky I., Cohen I.R., and Lider O. (1993). Extracellular matrix induces tumor necrosis factor -α secretion by an interaction between resting rat CD4+ T cells and macrophages. Immunology 78:50–57.

Kanazawa S., Ilic D., Noumura T., Yamamoto T., and Aizawa S. (1995). Integrin stimulation decreases tyrosine phosphorylation and activity of focal adhesion kinase in thymocytes. Biochem. Biophys. Res. Commun. 215:438–445.

Kitazawa H., Muegge K., Badolato R., Wang J.M., Fogler W.E., Ferris D.K., Lee C.K., Candeias S., Smith M.R., Oppenheim J.J., and Durum SK. (1997) IL-7 activates alpha4beta1 integrin in murine thymocytes. J. Immunol. 159:2259–2264.

Lagrota Cândido J.M., Vanderlei Jr. F.H., Villa Verde D.M.S., and Savino W. (1996). Exracellular matrix components of the mouse thymic microenvironment. V. Interferon-γ modulates thymocyte/thymic epithelial cell interactions via extracellular matrix ligands and receptors. Cell. Immunol. 170:235–244.

Lajoud M., Vremec D., Boyd R.L., and Shortman K. (1993). Characterization of thymic nurse cell lymphocytes, using an improved procedure for nurse cell isolation. Dev. Immunol. 3: 3:103–112.

Lannes Vieira J., Dardenne M., and Savino W. (1991). Extracellular matrix components of the mouse thymus microenvironment. I. Ontogenetic studies and modulation by glucocorticoid hormones. J. Histochem. Cytochem. 39:1539–1546.

Lannes Vieira J., Chammas R., Villa Verde D.M.S., Vannier Santos M.A., Souza S.J., Brentani R.R., and Savino W. (1993). Extracellular matrix components of the mouse thymus microenvironment. III. Thymic epithelial cells express laminin receptors that may modulate interactions with thymocytes. Int. Immunol. 5:1421–1430.

Leite-de-Moraes M.C., Hontebeyrie-Joskowicz M., Dardenne M., and Savino W. (1992). Modulation of thymocyte subsets during acute and chronic phases of experimental *Trypanosoma cruzi* infection. Immunology 77:95–98.

Li Y., Pezzano M., Philp D., Reide V., and Guyden, J. (1992). Thymic nurse cells exclusively bind and internalize $CD4^+CD8^+$ thymocytes. Cell. Immunol., 140:495–502.

Li Y.Q., Kobayashi M., Yuan L., Wang J., Matsushita K., Hamada J.I., Kimura K., Yagita H., Okumura K., and Hosokawa M. (1998). Protein kinase C mediates the signal for interferon-gamma mRNA expression in cytotoxic T cells after their adhesion to laminin. Immunology 93:455–461.

Lynch F., and Ceredig R. (1988). Ly-24 (Pgp-1) expression by thymocytes and peripheral T cells. Immunol. Today 9:7–10.

Magner W., Chang A.C., Owens J., Hong M.-J.P., Brooks A., and Coligan J.E. (1999). Aberrant development of thymocytes in mice lacking laminin-2. Dev. Immunol. – in press.

Meireles de Souza L.R., and Savino W. (1993). Modulation of cytokeratin expression in the hamster thymus. Evidence for a plasticity of the thymic epithelium. Develop. Immunol. 3:136–145.

Meireles de Souza L.R., Trajano V., and Savino W. (1993). Is there an inter-specific diversity of the thymic microenvironment? Develop. Immunol. 3:123–135.

Mello-Coelho V., Villa-Verde D.M.S., Dardenne M., and Savino. W (1997). Pituitary hormones modulate by extracellular matrix-mediated interactions between thymocyte and thymic epithelial cell mediated ligands and receptors. J. Neuroimmunol. 76:39–49.

Mizushima H, Koshikawa N, Moriyama K, Takamura H, Nagashima Y, Hirahara F, and Miyazaki K. (1998). Wide distribution of laminin-5 gamma 2 chain in basement membranes of various human tissues. Hormone Res. 50 Suppl 2:7–14.

Nieto M, Gomez M, Sanchez-Mateos P, Fernandez E, Marazuela M, Sacedon R, Varas A, Gonzalez-Amaro R, Zapata AG, Toribio ML, and Sanchez-Madrid F. (1996) Expression of functionally active alpha 4 beta 1 integrin by thymic epithelial cells. Clin. Exp. Immunol. 106:170–178.

Ocampo J.S.P., Brito J.M., Correa de Santana E., Borojevic R, Villa Verde D.M.S., and Savino W. (1999). Laminin isoforms modulate heterocellular interactions in the human thymus. Submitted to publication.

Ocklind G., Talts J., Fassler R., Mattsson A., and Ekblom P. (1993). Expression of tenascin in developing and adult mouse lymphoid organs. J. Histochem. Cytochem., 41:1163–1169.

Papiernik M., Nabarra B., Savino W., Pontoux C., and Barbey S. (1983). Thymic reticulum in mice. II. Culture and characterization of non-epithelial dendritic cells. Their role in the syngeneic stimulation of thymic medullary lymphocytes. Eur. J. Immunol., 13:147–155.

Patel D.D., Hale L.P., Whichard L.P., Radcliff G., Mackay C.R., and Haynes B.F. (1995). Expression of CD44 molecules and CD44 ligands during human thymic development: expression of CD44 isoforms is developmentally regulated. Int. Immunol. 7:277–286.

Ribeiro dos Santos R., Laus J.L., Silva J.S., Savino W., Rossi M., and Mengel J.O. (1992). Anti-CD4 abrogates rejection and re-establishes long-term tolerance to syngeneic newborn hearts grafted in mice chronically infected with *Trypanosoma cruzi*. J. Exp. Med. 175:29–39.

Ramarli D., Scupoli M.T., Fiorini E., Poffe O., Brentegani M., Villa A., Cecchini G., Tridente G., and Marchisio P.C. (1998). Thymocyte contact or monoclonal antibody-mediated clustering of $\alpha3\beta1$ or $\alpha6\beta4$ integrins activate interleukin-6 (IL-6) transcription factors (NF-kappaB and NF-IL6) and IL-6 production in human thymic epithelial cells. Blood 92:3745–2755.

Ruiz P., Wiles M.V., and Imhof B.A. (1995). Alpha 6 integrins participate in pro-T cell homing to the thymus. Eur. J. Immunol. 25:2034–2041.

Savino W., Villa Verde D.M.S., and Lannes Vieira J. (1993). Extracellular matrix proteins in intrathymic T cell migration and differentiation? Immunol. Today 14:158–161.

Savino W., Leite de Moraes M.C., Hontebeyrie-Joskowicz M., and Dardenne M. (1989). Studies on the thymus in Chagas' disease. I. Changes in the thymic microenvironment in mice acutely infected with Trypanosoma cruzi. Eur. J. Immunol., 19:1727–1733.

Savino W., Boitard C., Bach J.F., and Dardenne M. (1991). Studies on the thymus in nonobese diabetic (NOD) mice. I. Changes in the microenvironmental compartments. Lab. Invest. 64:405–417.

Savino W., Carnaud C., Luan J.J., Bach J.F., and Dardenne M. (1993). Further characterization of the extracellular matrix-containing giant perivarcular spaces in the thymus of the nonobese diabet mouse. Diabetes 42:134–140.

Savino W., Dardenne M., and Carnaud C. (1996). Conveyor belt hypothesis for intrathymic cell migration: possible relationship with extracellular matrix. Immunol. Today 17:292–293.

Savino W, Dardenne W, and Lepault F (1998). The thymic extracellular matrix in genetically engineered mice. Immunol. Today 19:144–145.

Sawada M., Nagamine J., Takeda K., Utsumi K., Kosugi A., Tatsumi Y., Hamaoka T., Miyade K., Nakajima K., Watanbe T., Sakakibara S., and Fujiwara, H. (1992) Expression of VLA-4 on thymocytes. Maturation stage-associated transition and its correlation with their capacity to adhere to thymic stromal cells. J. Immunol. 149:3517–3524.

Silva Barbosa S.D., Cotta de Almeida V., Riederer I., Dardenne M. Bonomo A.C., and Savino W. (1997). Involvement of laminin and its receptor upon syngeneic heart graft rejection by $CD4^+$ autoreactive lymphocytes derived from *Trypanosoma cruzi*-chronically-infected mice. J. Immunol. 159:997–1003.

Sawada M., Nagamine J., Takeda K., Utsumi K., Kosugi A., Tatsumi Y., Hamoaka T., Miyake K., Nakajimo K., Watanabe T., Sakakibara S., and Fujiwara, H. (1992). Expression of VLA-4 on thymocytes. Maturation stage-associated transition and its correlation with their capacity to adhere to thymic stromal cells. J. Immunol. 149:3517–3524.

Uniyal S., Boeters L., and Chan B.M.C. (1998) A comparison in $\beta1$ integrin expression during thymocyte maturation in Balb/c and nonobese diabetic (NOD) mice. $10^{th}$ International Congress of Immunology (New Delhi). The Immunologist (suppl. 1): 255.

Utsumi K., Sawada M., Narumiya S., Nagamine J., Sakata T., Iwagami S., Kita Y., Teraoka H., Hirano H., Ogata M., Hamaoka T., and Fujiwara H. (1991). Adhesion of immature thymocytes to thymic stromal cells through fibronectin molecules and its significance for the induction of thymocyte differentiation. Proc. Natl. Acad. Sc. USA 88:5685–5689.

Takayama E, Kina T, Katsura Y, and Tadakuma T. (1998). Enhancement of activation-induced cell death by fibronectin in murine $CD4^{+}CD8^{+}$ thymocytes. Immunology 95: 553–558.

Tchilian E.Z., Owen J.J.T., and Jenkinson E.J. (1997). Anti-$\alpha 4$ integrin antibody induces apoptosis in murine thymocytes and staphylococcal enterotoxin B-activated lymph node T cells. Immunology 92:321–327.

van Ewijk W. (1988). Cell surface topography of thymic microenvironments. Lab. Invest. 59:579–587.

Villa Verde D.M.S., Chammas R., Lagrota Candido J.M., Brentani R.R., and Savino W. (1994). Extracellular matrix components of the mouse thymus microenvironment. IV. Thymic nurse cells express extracellular matrix ligands and receptors. Eur. J. Immunol., 24:659–664.

Villa Verde D.M.S., Mello-Coelho V., Lagrota-Cândido J.M., and Savino W. (1995). The thymic nurse cell complex: an *in vitro* model for extracellular matrix-mediated intrathymic T cell migration. Braz. J. Med. Biol. Res. 28:907–912.

Villa-Verde D.M.S., Calado T.J., Ocampo J.S.P., and Savino W (1999). Coveyor belt hypothesis for intrathymic T cell migration: role of adhesion and de-adhesion molecules Braz. J. Med. Biol. Res. 32:569–572.

Vivinus-Nebot M., Ticchioni M., Mary F., Hofman P., Quaranta V., Rousselle P., and Bernard A. (1999) Laminin 5 in the human thymus: control of T cell proliferation via $\alpha 6\beta 4$ integrins. J. Cell Biol. 144:563–574.

Wadsworth S., Halvorson M.J., and Coligan J.E. (1992). Developmentally regulated expression of the $\beta 4$ integrin on immature mouse thymocytes. J. Immunol. 149:421–426.

Wekerle H., and Ketelsen U.P. (1980). Thymic nurse cells. Ia-bearing epithelium involved in T-lymphocyte differentiation. Nature 283:402–403.

Werneck C.C., Oliveira-dos-Santos A.J., Silva L.C.F., Villa-Verde D.M.S., Savino W., and Mourão P.A.S. (1999). Thymic epithelial cells synthetize a heparan sulfate with short but highly sulfated regions. J. Cell. Physiol. 178:51–62.

Zaitseva M.B., Mojcik C.F., Salomon D.R., Shevach E.M., and Golding H. (1998) Coligation of alpha4beta1 integrin and TCR rescues human thymocytes from steroid-induced apoptosis. Int. Immunol. 10:1551–1561.

